# Moderate alcohol consumption during pregnancy increases potency of two different drugs (the antifungal fluconazole and the antiepileptic valproate) in inducing craniofacial defects: prediction by the in vitro rat whole embryo culture

**DOI:** 10.1007/s00204-022-03410-2

**Published:** 2022-11-16

**Authors:** Francesca Metruccio, Maria Battistoni, Francesca Di Renzo, Angelo Moretto, Elena Menegola

**Affiliations:** 1 ICPS, ASST Fatebenefratelli Sacco, via GB Grassi, 74, 20159 Milan, Italy; 2 Department of Physics Aldo Pontremoli, Universita` degli Studi di Milano, via Celoria, 16, 20133 Milan, Italy; 3 Department of Environmental Science and Policy, Universita` degli Studi di Milano, via Celoria, 26, 20133 Milan, Italy; 4 Department of Cardiac Thoracic Vascular and Public Health Science, Università degli Studi di Padova, via Giustiniani 2, 35128 Padua, Italy

**Keywords:** Pregnancy, Embryo, Mixture, Ethanol, Antifungal, Antiepileptic

## Abstract

The prenatal exposure to ethanol (Eth), fluconazole (FLUCO) and sodium valproate (VPA) is related to effects on development, producing characteristic syndromic pictures. Among embryotoxic effects described for the three molecules, the alteration on craniofacial morphogenesis is a common feature in humans and animal models, including rodent embryos developed in vitro. The aim of the present work is to evaluate the developmental effects of low Eth serum concentration (17 mM, corresponding to the legal limit to drive in UK, USA, Canada, and many other countries) in mixture with increasing realistic concentrations of the antifungal drug FLUCO (62.5–500 µM) or with increasing realistic concentrations of the antiepileptic drug VPA (31.25–250 µM). Groups exposed to Eth alone (17–127.5 mM), FLUCO alone (62.5–500 µM) or VPA alone (31.25–750 µM) were also included. The chosen alternative animal model was the post-implantation rat whole embryo culture (WEC). E9.5 embryos were exposed in vitro to the test molecules during the whole test period (48 h, corresponding to the developmental stages characteristics of any vertebrate, for human embryos post-fertilization days 23–31). Data were statistically analyzed and processed for modelling applying the benchmark dose (BMD) and relative potency factor (RPF) approaches. Concentration-related effects on facial outcomes were observed in all experimental groups, with a significant enhancement in the groups co-exposed with Eth in comparison to the single exposures. Data obtained by the present work suggest an additional alert for the assumption of even low levels of alcohol in pregnant women during FLUCO or VPA therapy.

## Introduction

Alcohol abuse in pregnancy causes a wide range of adverse effects in newborns with severity depending on several factors which include the timing, pattern, and dose of consumption (Caputo et al. 2016). The alcohol-related spectrum of physical, cognitive, and behavioral disabilities in newborns is known as fetal alcohol spectrum disorder (FASD) (Sulik 2005; Kotch and Sulik 1992; Willford et al. 2006). The most severe form, that includes morphological abnormalities, is defined as fetal alcohol syndrome (FAS) (de Sanctis et al. 2011; Joya et al. 2012; Memo et al. 2013). FAS babies show neurocognitive disorders, growth deficits, and typically identifiable craniofacial habitus (microcephaly, flat midface with short palpebral fissures, low nasal bridge with short nose and long smooth or flat philtrum) (de Sanctis et al. 2011) and 50% of affected children also exhibit malformations (cleft palate, maxillary hypoplasia, and micrognathia) (Jacobs 2000) The collective evidence from human and animal studies strongly suggests that even light drinking during pregnancy can produce significant neuropsychological long-lasting alterations (Flak et al. 2014) and anatomical variations in craniofacial shape (Muggli et al. 2017). In spite of WHO alert for women of childbearing age to avoid alcohol consumption, in the occidental Western regions a large proportion of women drinks alcohol at least till evidence of pregnancy. Considering that approximately half of all pregnancies is unplanned and a significant percentage of women continues to drink alcoholic beverages during pregnancy, in utero exposure to alcohol is a public health concern (Meurk et al. 2014; Schölin 2016).

In addition to Eth, antimycotics and antiepileptics drugs assumed in pregnancy are included in etiological factors of craniofacial defects (Jentink et al. 2010; Howley et al. 2016; Foster and Patel 2019). Considering that the combined exposure to alcohol and drugs is not a rare event (Foster and Patel 2019), the issue of an impact on newborn risk due to the co-exposure in pregnancy to drugs and alcohol is an under-investigated concern.

The present work intends to evaluate the developmental effects of physiologically relevant ethanol (Eth) concentration (17 mM, corresponding to the legal limit to drive in UK, USA, Canada, and many other countries) in combination with the antimycotic fluconazole (FLUCO) or with the antiepileptic sodium valproate (VPA). This was carried out by using the rat post-implantation whole embryo culture (WEC) method.

WEC is an ECVAM validated alternative method intended to identify substances which induce malformation resulting in embryotoxicity (ECVAM 2006). WEC method enabled detailed studies on normal/abnormal embryo development as well as researches on mechanisms of chemical-induced teratogenesis (Ellis-Hutchings and Carney 2010).

This in vitro procedure allows to expose embryos at the phylotypic stage with precise control of exposure parameters and, removing confounding maternal pharmacokinetics/pharmacodynamics variables, enables to avoid maternal species-specificity. The general concept of phylotypic stages states that, among an evolutive group, early embryos resemble each other developing key structures by conserved molecular expression patterns. In vertebrates, early morphogenesis leads to transitory common structures (dorsal neural tube with encephalic vesicles, trunk segmental somites, segmented pharyngeal branchial arches) responsible for the adult body plan (defined as a set of morphological traits shared by any vertebrate, from fishes to humans: overt head, trunk with segmented vertebrae and a segmented pharynx) (Duboule 1994; Irie and Kuratani 2011).

Using WEC, Eth, FLUCO and VPA induce severe developmental defects, including abnormalities at the embryonic precursors of facial skeleton (branchial arches): Eth concentrations of 44 mM or higher are teratogenic, while 17 mM is universally accepted as ineffective and therefore used in WEC as solvent for water-insoluble test molecules (Kitchin and Ebron 1984; Fadel and Persaud 1992; Giavini et al. 1992; Zhou et al. 2011); FLUCO is teratogenic at same order of magnitude concentrations (125–500 µM) (Tiboni 1993; Menegola et al. 2001) of the therapeutical plasma level window (13–228 µM) (Santos et al. 2010); VPA-exposure related to developmental defects at concentrations (31.25–750 µM) (Metruccio et al. 2020; Battistoni et al. 2022) consistent with plasma therapeutic levels (347–693 µM or higher) (Turnbull et al. 1983; Nakashima et al. 2015).

The aims of the present work are: (i) to describe dose–response curves for single compounds (Eth, FLUCO or VPA) and mixtures (fix and moving protocol, Eth 17 mM + increasing concentrations of FLUCO and Eth 17 mM + increasing concentrations of VPA); (ii) to apply the benchmark dose (BMD) modelling approach to calculate relative potency factors (RPFs) describing the influence of a low Eth concentration on FLUCO or VPA effects.

## Materials and methods

### Materials and compound preparation

Eth (Fluka, purity ≥ 99.5%), FLUCO (Sigma, purity ≥ 98%), VPA (Sigma, purity ≥ 97.5%) were used as test substances. FLUCO and VPA were dissolved in distilled water to obtain mother solutions (FLUCO 10 mg/1.3 mL; VPA: 10 mg/0.8 mL) and then subsequently diluted in physiological Tyrode solution (Sigma) to obtain working solutions, Eth undiluted was diluted in Tyrode to obtain working solutions. The final total added volume was 20 µL/mL culture medium. The culture medium was undiluted heat inactivated rat serum (obtained according to (Menegola et al. 1995)) added with antibiotics (penicillin 100 IU/mL culture medium and streptomycin 100 µg/mL culture medium, Sigma).

### Selection of compound concentrations

The dose–response curves for the single molecules were derived selecting concentrations from previous published experiments: FLUCO 0–62.5–125–250–500 µM (Menegola et al. 2001), Eth 0–17–42.5–85–127.5 mM (Priscott 1982; Wynter et al. 1983; Kitchin and Ebron 1984; Clode et al. 1987; Giavini et al. 1992; Hunter et al. 1994; van Maele-Fabry et al. 1995), VPA 0–31.25–62.5–125–250–375–500–750 µM (Metruccio et al. 2020; Battistoni et al. 2022). Mixture effect evaluation was performed following the fix and moving design. FLUCOmix groups: Eth fix (17 mM) + FLUCO moving (0–62.5–125–250–500 µM); VPAmix groups: Eth fix (17 mM) + VPA moving (0–31.25–62.5–125–250 µM).

### Animals

Protocols involving animal use were approved by the Ministry of Health- Department for Veterinary Public Health, Nutrition and Food Safety committee. Animals were treated humanely and with regard for alleviation of suffering. All applicable international, national, and/or institutional guidelines for the care and the use of animals were followed. Animals were kept in pathogen-free/controlled conditions and all procedures were authorized by Ministry permission in compliance with Italian law (D. Lgs no. 2014/26, implementation of the 2010/63/UE).

Virgin female CD:Crl rats (Charles River, Calco, Italy), housed in a thermostatically maintained room (*T* = 22 ± 2 °C; relative humidity 55 ± 5%) with a 12 h light cycle (light from 6.00 a.m. to 6.00 p.m.), free access to food (Italiana Mangimi, Settimo Milanese, Italy) and tap water ad libitum, were caged overnight with males of proven fertility. Dams with positive vaginal smear at the morning after were considered pregnant (day of positive vaginal smear = E0).

### Embryo culture

Embryos were explanted from different pregnant rats at E9.5 (early neurula stage, 1–3 somites), randomly distributed into the experimental groups and cultured according to the New’s method (NEW 1978) in 20 mL glass bottles (5 embryos/bottle), containing 5 mL culture medium. At least a triplicate was performed for each group. The bottles, inserted in a thermostatic (37.8 °C) roller (30 rpm) apparatus, were periodically gas equilibrated according to Giavini et al. (1992). Briefly, the culture medium was 1 min equilibrated with 1 bar gas flow (Siad, Italy) every 12 h with increasing O_2_ content (5–10–20–20%), decreasing N_2_ and fixed 5% CO_2_. After 48 h of culture, embryos were morphologically examined under a dissecting microscope and abnormalities were recorded and classified into two categories: branchial abnormalities and any other developmental defects. Features examined were those described by Brown and Fabro (1981).

### Statistics

To evaluate dose–response relationship, data sets obtained in the different concentration groups for each exposure condition (Eth, FLUCO, FLUCOmix, VPA, VPAmix) were statistically analyzed using extended Mantel Haenszel chi-square test for trend. FLUCO/FLUCOmix and VPA/VPAmix paired comparisons were carried out on each concentration, applying the two tailed exact Fisher test. The level of significance was set at *p* < 0.05.

### Data modelling

The benchmark dose (BMD) approach was applied using PROAST (70.3 version). The BMD approach involves a statistical method, which uses the information in the complete dataset instead of making pair-wise comparisons using subsets of the data. In addition, the BMD approach can interpolate between applied doses, with the evaluation of the uncertainty in the calculated BMD, which is reflected by the confidence interval around the BMD. PROAST is a software package specifically developed by the Dutch National Institute for Public Health and the Environment (RIVM) (www.proast.nl) for modelling dose–response data. Data were modelled to characterize each dose response curve and to calculate benchmark doses (BMD_50_) (i.e., the doses estimated to cause 50% response, BMR_50_). To compare the different dose–response curves obtained for each molecule alone or combined with Eth, the loglikelihood ratio test was applied to assess the equal steepness assumption. Once the test was passed, the potency comparison was made and the relative potency factors (RPFs) of FLUCOmix versus FLUCO and of VPAmix versus VPA derived.

## Results

### Effects on Eth, FLUCO, VPA, FLUCOmix and VPAmix groups

**Fig. 1:**
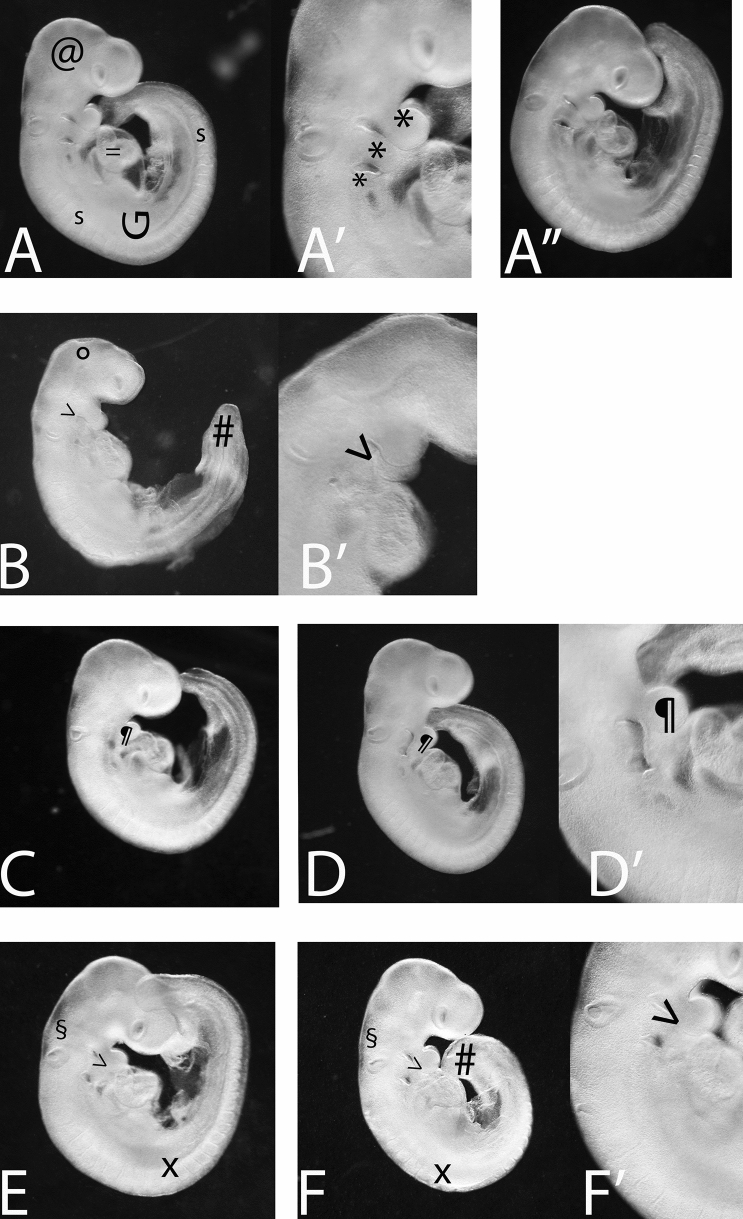
Morphology of embryos at the end of WEC. Magnification 20X (**A**–**F**, **A**’’), 40X (**A**’–**F**’). **A**–**A**’ Eth 17 mM embryo showing a normal morphology. Note the head with the encephalon (@) and three separated branchial arches (***). The embryo appears dorsally convex, with a G-shaped axis (G) characterized by regular somites (s). The concamerated heart is visible ventrally ( =). **A**’’ Unexposed control embryo showing the same morphological characteristics described in (**A**–**A**’). **B**–**B**’ Eth 42.5 mM embryo with reduced branchial arches ( >) and abnormalities affecting the encephalon (microcephalia with open neural tube, °) and the axial structures (defects classified as hook-shaped tail, #). **C** FLUCO 500 µM with branchial defects (fused branchial arches, ¶). **D**–**D**’ FLUCO 250 µM in mixture with Eth 17 mM embryo showing branchial defects (fused branchial arches, ¶) similar to those observed in embryos exposed to higher concentration of FLUCO alone (**C**). **E** VPA 375 µM showing branchial abnormalities (fused branchial arches, >) and other developmental defects (swollen encephalon, §; axial defects: hook-shaped tail, # or irregular and fused somites, X) abnormalities. **F**–**F**’ VPA 250 µM in mixture with Eth 17 mM embryo showing abnormalities (fused branchial arches, > ; swollen encephalon, §; axial defects: hook-shaped tail, # or irregular and fused somites, X) similar to those observed in embryos exposed to higher concentration of VPA alone (**E**)

After 48 h in culture, normal embryos displayed the typical characteristic of any vertebrate embryo (including human embryos) at the phylotypic stage: dorsally convex, dorsal neural tube with enlarged encephalic ventricles, three well separated branchial arches (the embryonic precursors of facial structures), concamerated ventral heart, somites (the precursors of axial elements) (Fig. 1A–A’–A’’). Teratogenic effects were detected in embryos exposed to tested molecules: Eth, FLUCO, VPA and mixture groups (Tables 1, 2 and 3; Fig. 1B–F, B’–F’). A syndromic picture, including both branchial (branchial arch fused or reduced in size) and other developmental defects (encephalic and axial abnormalities), was observed in Eth or VPA groups (Tables 1 and 3; Fig. 1B–B’, E–F–F’), while only branchial defects characterized FLUCO and FLUCOmix groups (Table 2; Fig. 1C–D–D’). Two-by-two Fisher exact statistical comparison was performed to detect, for each dose, significant differences between single-compound and Eth mixture. Statistical differences were evident only for branchial defects at the lower FLUCO concentration (Table 2) and at VPA concentrations ≥ 62.5 µM (Table 3). VPA related encephalic and axial abnormalities were never increased in mixture groups (Table 3).

**Table 1:** Branchial and extra-branchial abnormalities induced by Eth, evaluated at the end of the culture period

	Eth 0 mM	Eth 17 mM	Eth 42.5 mM	Eth 85 mM	Eth 127.5 mM	*p* (chi-square for trend)
Branchial abnormalities (branchial arch fused or reduced in size) (%)	0	0	72.7	80.0	100	** < o.oooooo1**
Other developmental defects (encephalic and axial abnormalities) (%)	0	10	27.27	53.33	100	** < o.oooooo1**

To evaluate dose–response relationship, data sets obtained in the different concentration groups were statistically analyzed using extended Mantel Haenszel chi-square test for trend. Data displayed as percentages, statistics performed on frequencies. Grey: data on concentration used in mixture groups

**Table 2:** Branchial and extra-branchial abnormalities induced by FLUCO and FLUCOmix (FLUCO in presence of Eth 17 mM) evaluated at the end of the culture period

		FLUCO 0 µM	FLUCO 62.5 µM	FLUCO 125 µM	FLUCO 250 µM	FLUCO 500 µM	*p* (chi-square for trend)
Branchial arch abnormalities (branchial arch fused or reduced in size) (%)	**Eth 0 mM**	0	0	44.4	72.2	100	** < o.oooooo1**
	**Eth 17 mM**	0	47.4**	72.2	94.4	100	** < o.oooooo1**
Other developmental defects (encephalic and axial abnormalities) (%)	**Eth 0 mM**	0	0	0	0	0	**_**
	**Eth 17 mM**	0	10	0	0	0	**_**

Grey: data on mixture groups; **p < 0.01 FLUCOmix vs. the group exposed to FLUCO alone at the same concentration. To evaluate dose–response relationship, data sets obtained in the different concentration groups were statistically analyzed using extended Mantel Haenszel chi-square test for trend. FLUCO/FLUCOmix paired comparisons were carried out on each concentration, applying the two tailed exact Fisher test. Data displayed as percentages, statistics performed on frequencies

**Table 3:** Branchial and extrabranchial abnormalities induced by VPA and VPAmix (VPA in presence of Eth 17 mM) evaluated at the end of the culture period

		VPA 0 µM	VPA 31.25 µM	VPA 62.5 µM	VPA 125 µM	VPA 250 µM	VPA 375 µM	VPA 500 µM	VPA 750 µM	*p* (chi-square for trend)
Branchial arch abnormalities (branchial arch fused or reduced in size) (%)	**Eth 0 mM**	0	25.0	17.6	43.8	36.4	50.0	50.0	80.0	**0.00017**
	**Eth 17 mM**	0	7.7	75.0**	88.9*	77.8*				** < o.oooooo1**
Other developmental defects (encephalic and axial abnormalities) (%)	**Eth 0 mM**	6.3	44.4	50.0	57.1	50.0	38.5	41.7	100	**0.029**
	**Eth 17 mM**	0	23.1	66.7	33.3	77.8				**0.00001**

Grey: data on mixture groups; *p < 0.05 VPAmix vs. the group exposed to VPA alone at the same concentration. **p < 0.01 VPAmix vs. the group exposed to VPA alone at the same concentration

To evaluate dose–response relationship, data sets obtained in the different concentration groups were statistically analyzed using extended Mantel Haenszel chi-square test for trend. VPA/VPAmix paired comparisons were carried out on each concentration, applying the two tailed exact Fisher test. Data displayed as percentages, statistics performed on frequencies

### Data modelling

**Fig. 2:**
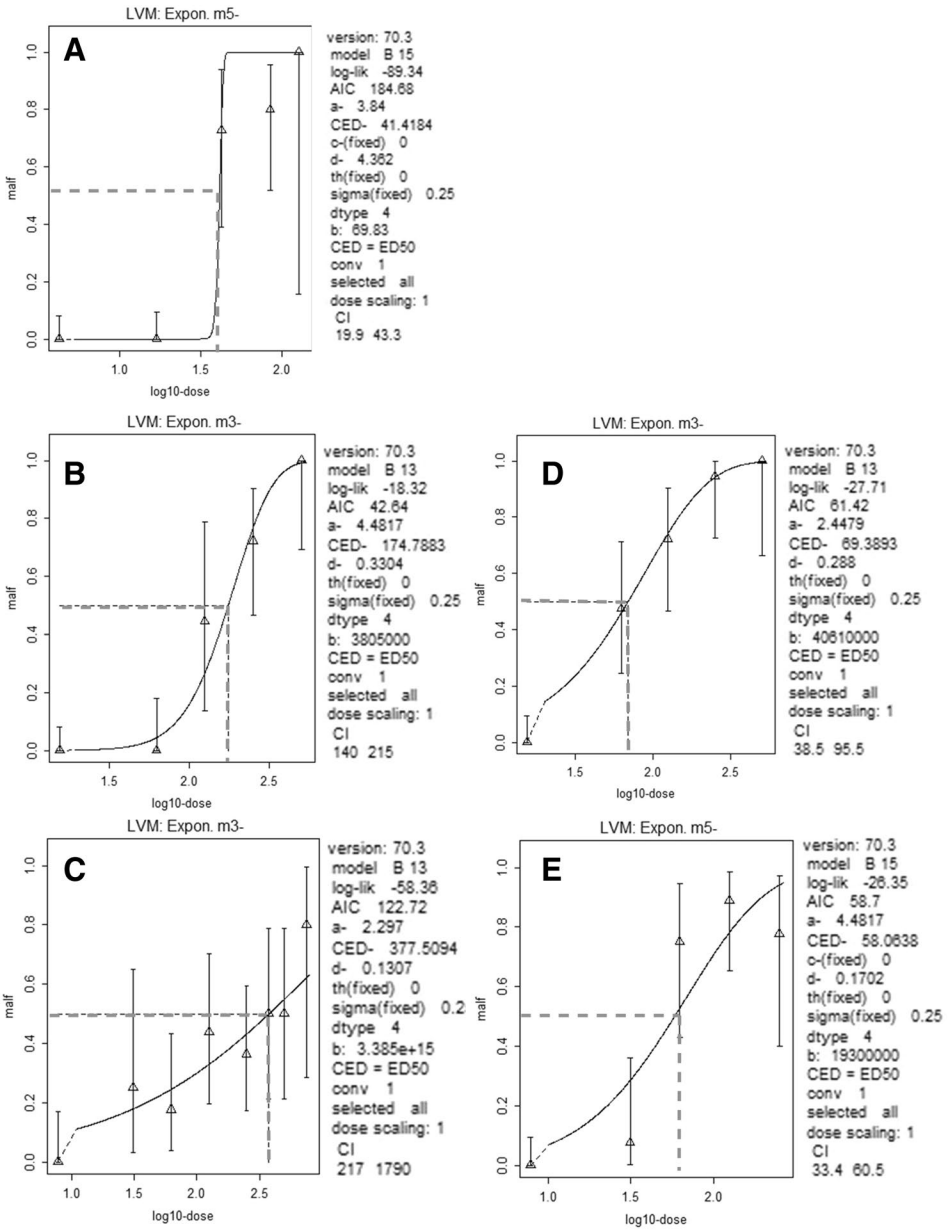
Single dose–response curves of Eth (**A**), FLUCO (**B**), VPA (**C**), FLUCOmix (**D**), VPAmix (**E**). Dose is plotted in log-scale. Response = fraction of abnormal embryos. Dotted lines = BMD for 50% BMR. Vertical whiskers represent 95% Confidence Intervals for each response data point

**Fig. 3:**
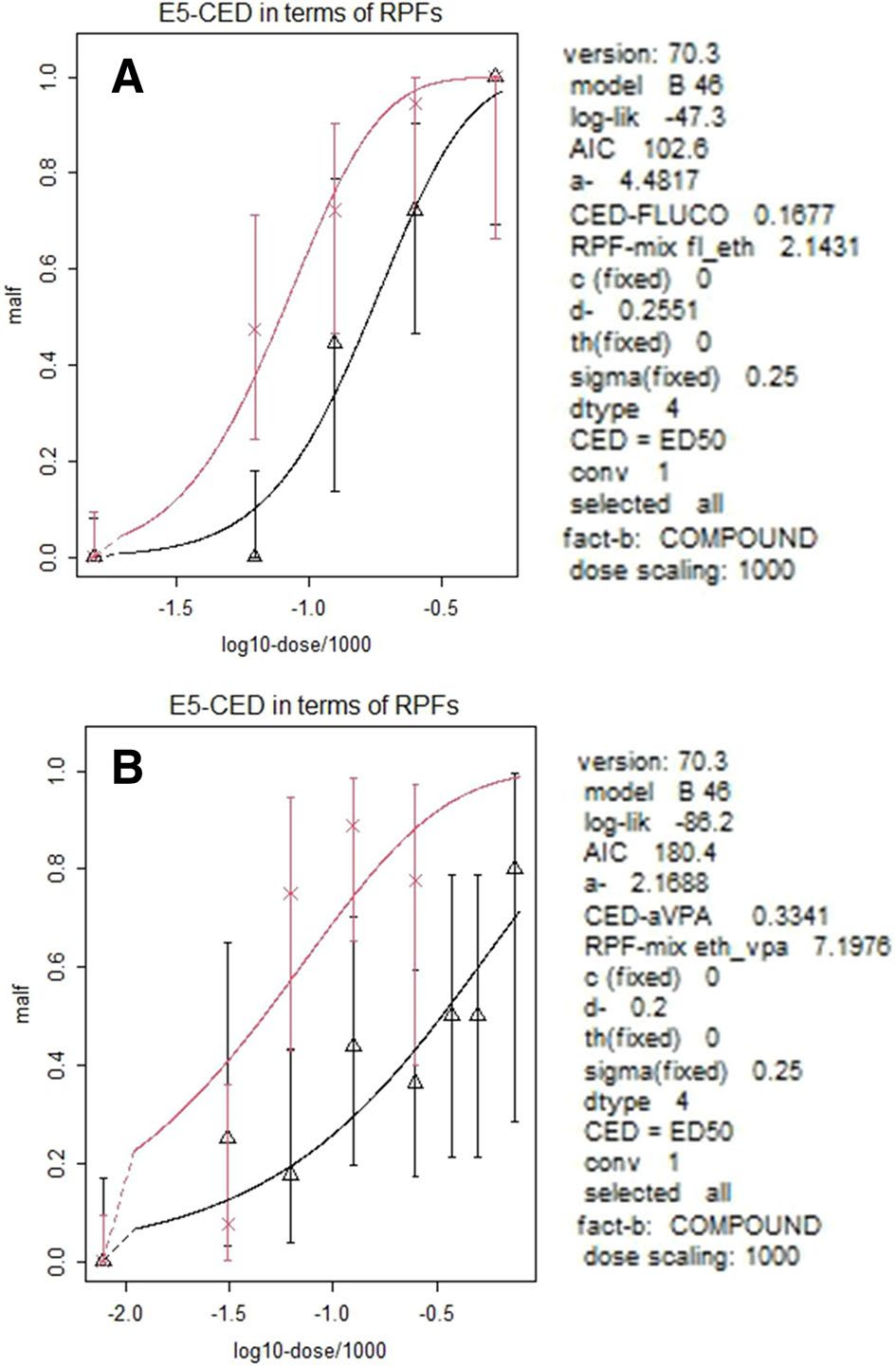
Evaluation of the relative potency factors (RPFs) of the effects of mix versus single compound. **A** FLUCO vs. FLUCOmix, **B** VPA vs VPAmix. Red line/cross data points indicate mixture, black line/triangles data point single compound curves. Dose is plotted in log-scale/1000. Response = fraction of abnormal embryos. Vertical whiskers represent 95% Confidence Intervals for each response data point

**Fig. 4:**
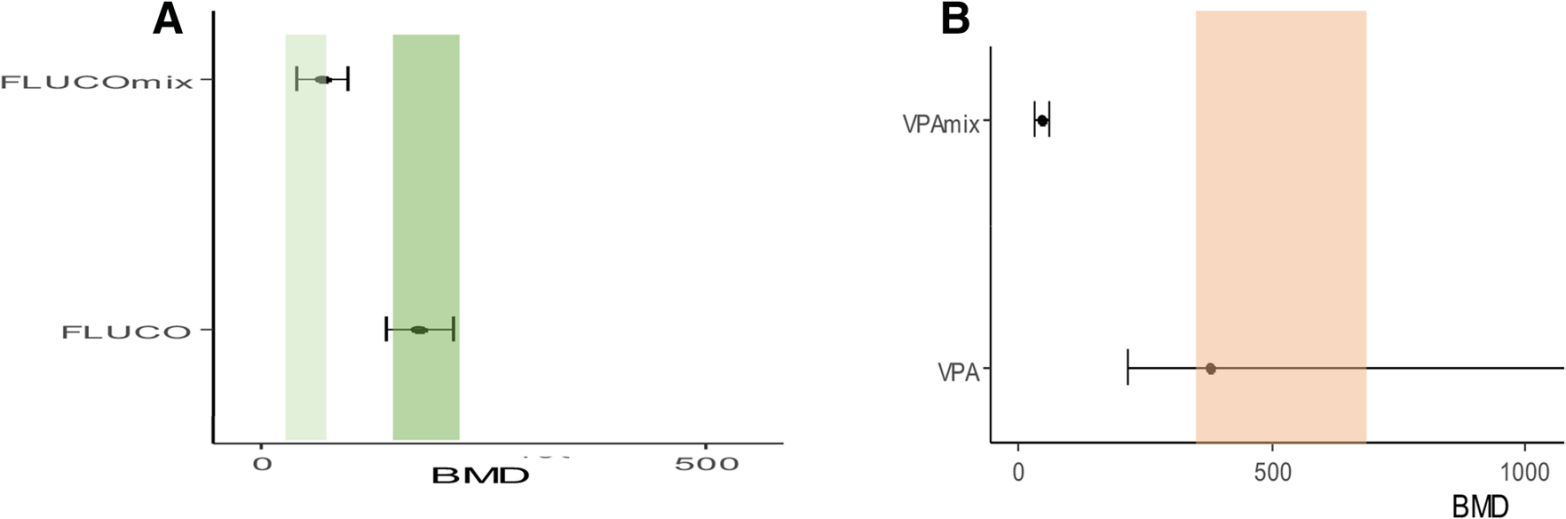
**A** Plot of benchmark doses (BMDs) for benchmark response at 50% with CIs for FLUCO alone and in mixture with Eth (FLUCOmix). Light rectangle represents the plasma concentration range reached with low dose FLUCO therapeutical regimen (related to FDA classification C), dark rectangle represents the plasma concentration range reached with high dose FLUCO therapeutical regimen (related to FDA classification D); **B** Plot of benchmark doses (BMDs) for benchmark response at 50% with CIs for VPA alone and in mixture with Eth (VPAmix). Rectangle represents the plasma concentration range reached with VPA therapeutical regimen (related to FDA classification D)

Only outcomes with a positive linear trend were modelled to obtain BMD_50_ (Table 4). Due to the fact that encephalic and axial defects (other defects) were statistically significant only in Eth and VPAmix groups, the following analyses were performed only on branchial outcomes: (i) the single dose response curves in the different experimental groups were characterized (Fig. 2); (ii) BMD_50_ CIs obtained for each Eth mixture compared to its relative compound do not overlap, and this evidence supports a difference in potency verified by relative potency factors (RPFs) estimation (Table 5; Fig. 3). RPF CIs showed that, in both cases, mixtures are more effective than single molecules. At the tested concentrations, this appeared more marked for VPAmix/VPA (resulting in RPF 7.2, Fig. 3A), while FLUCOmix/FLUCO RPF was 2.1 (Fig. 3B). Figure 4 shows the plots of BMDs for BMRs 50% obtained for branchial outcomes in the different experimental groups. Interestingly, these concentrations overlap or are lower than the realistic human plasma concentrations.

**Table 4:** Benchmark Doses (BMDs) for 50% Benchmark Responses (BMRs) with 95% Confidence Intervals (BMDL-BMDH) calculated in groups showing a significant linear trend for the considered outcome

Group	BMD	BMDL	BMDH
Branchial defects			
Eth	41.4	19.9	43.3
FLUCO	174.8	140.0	215.0
FLUCOmix	69.4	38.5	95.5
VPA	377.5	217.0	1790.0
VPAmix	47.4	33.4	60.5
Other defects			
Eth	72.4	52.3	126.0
VPAmix	110.1	39.2	11,700.0

Eth mM, FLUCO and VPA µM

**Table 5:** Relative Potency Factors (RPFs) with 95% Confidence Intervals (RPFL-RPFH) between FLUCOmix versus FLUCO and between VPAmix versus VPA

Group	RPF	RPFL	RPFH
Branchial defects			
FLUCO	1		
FLUCOmix	2.1	1.6	3.1
VPA	1		
VPAmix	7.2	3.8	16.6

## Discussion

First aim of the present work was the evaluation of Eth, FLUCO and VPA-related teratogenic effects using the in vitro post-implantation rat whole embryo culture method (WEC).

WEC is proposed as a good model for human teratogenic hazard prediction. It is relevant to notice that, at the considered embryonic stages, the definitive placenta is not yet developed and consequently, in humans as in rodents, the placenta does not influence the maternal–fetal transports (Burton and Jauniaux 2018; Knöfler et al. 2019). Excluding any maternal influence, WEC is a species-agnostic test (i.e., the embryonic response can be transversally extrapolated to any vertebrate, from fishes to humans). This is because all vertebrate embryos at early developmental stages (phylotypic stages) are identical both at the morphological and molecular levels (Irie and Kuratani 2011). This means that the blueprint of body formation is defined by the same molecular basis in all vertebrates and any change on developmental phylotypic processes will alter the embryogenesis of different species in a similar way. WEC embryonic stages overlap with phylotypic stages and correspond to human post-fertilization days 23–31 (http://virtualhumanembryo.lsuhsc.edu/heirloom/Stages/Stages_intro.html). During this organogenetic period, before the chorionic circulation is fully established at human 12 weeks of gestation, the definitive placenta is not yet developed and, similarly to rodents, the human yolk sac vascularization plays a key role in maternal–fetal transport. (Burton and Jauniaux 2018; Knöfler et al. 2019). A full correspondence between human/WEC conditions, however, remains impossible due to different species-specific and individual maternal metabolic factors characterizing the in vivo exposure.

In our experiments Eth, FLUCO and VPA were able to affect embryonic key structures (branchial arches, encephalon, axial structures) precursors of the typical vertebrate traits. The described embryonic abnormalities can be predictive for human defects when embryos are exposed during the first month of pregnancy.

FLUCO can be dosed orally (tablets and suspension) or by intravenous infusion. As indicated by producers, since oral absorption is rapid and almost complete, the daily dose of FLUCO is the same for oral and intravenous administration (150 mg/day for uncomplicated vaginal candidiasis, 200 mg/day for oropharyngeal and esophageal candidiasis, 400–800 mg/day for systemic mycoses). Effective maternal plasma concentrations range from 20 to 230 mM (Mikamo et al. 1999; Santos et al. 2010). VPA is intended for oral administration, in capsules or oral solution. The usual dose for treating epilepsy is 600–2500 mg/day, for treating bipolar disorder is 750–2000 mg/day, for preventing migraine is 400–1500 mg/day. Effective maternal plasma concentrations range from 347 to 693 mM (Turnbull et al. 1983; Nakashima et al. 2015). In the present work, Eth, BMD for BMR 50% resulted in the range of plasma concentrations reached after moderate/mild alcohol consumption (https://alcohol.org/effects/blackouts-dangers/) and both FLUCO and VPA BMDs for BMR 50% fall in therapeutic plasma concentration ranges (Turnbull et al. 1983; Mikamo et al. 1999; Santos et al. 2010; Nakashima et al. 2015) (Fig. 4). Our findings are in line with the WHO alert of possible adverse effects due to Eth consumption at any dosage and with FDA assignment of VPA and high-dose regimen FLUCO to pregnancy category D (drugs which have caused, are suspected to have caused or may be expected to cause, an increased incidence of human fetal malformations or irreversible damage) (Zawab and Carmody 2014; Pilmis et al. 2015). By contrast, nowadays FLUCO falls into the FDA category C (risk cannot be ruled out) if assumed at low dose regimen (Pilmis et al. 2015).

Our data suggest a reclassification of FLUCO in the case of moderate drinking pregnant women. In fact, in spite of FLUCO leaflets indicating to avoid FLUCO therapy in pregnancy and to use contraceptive measures during treatment in fertile women, low-dose regimen FLUCO treatment represents a short-term therapy prescribed by general physicians, pharmacists and, in several countries, also by self-prescription. Moreover, FLUCO leaflets or medical blogs do not mention additional risk in pregnancy when moderate alcohol is consumed. However, our data show a significant left-shift of the dose–response curve for teratogenic effects when Eth is co-assumed. Finally, despite its teratogenic characteristics, the use of FLUCO during early pregnancy (when the woman is not yet aware to be pregnant) is a realistic possibility. Our results on Eth mixtures provide also the first evidence that Eth at low concentration markedly increases VPA potency in eliciting branchial defects. It has to be underlined that VPA chronic therapeutic regimen is prescribed by specialists who are well aware for VPA teratogenic effects and for the need to avoid VPA therapy, unless essential, in women of childbearing age. In the case of VPA medication during pregnancy, physicians and patients should be aware on possible additional risk of malformations due to low/moderate alcohol consumption.

**Fig. 5:**
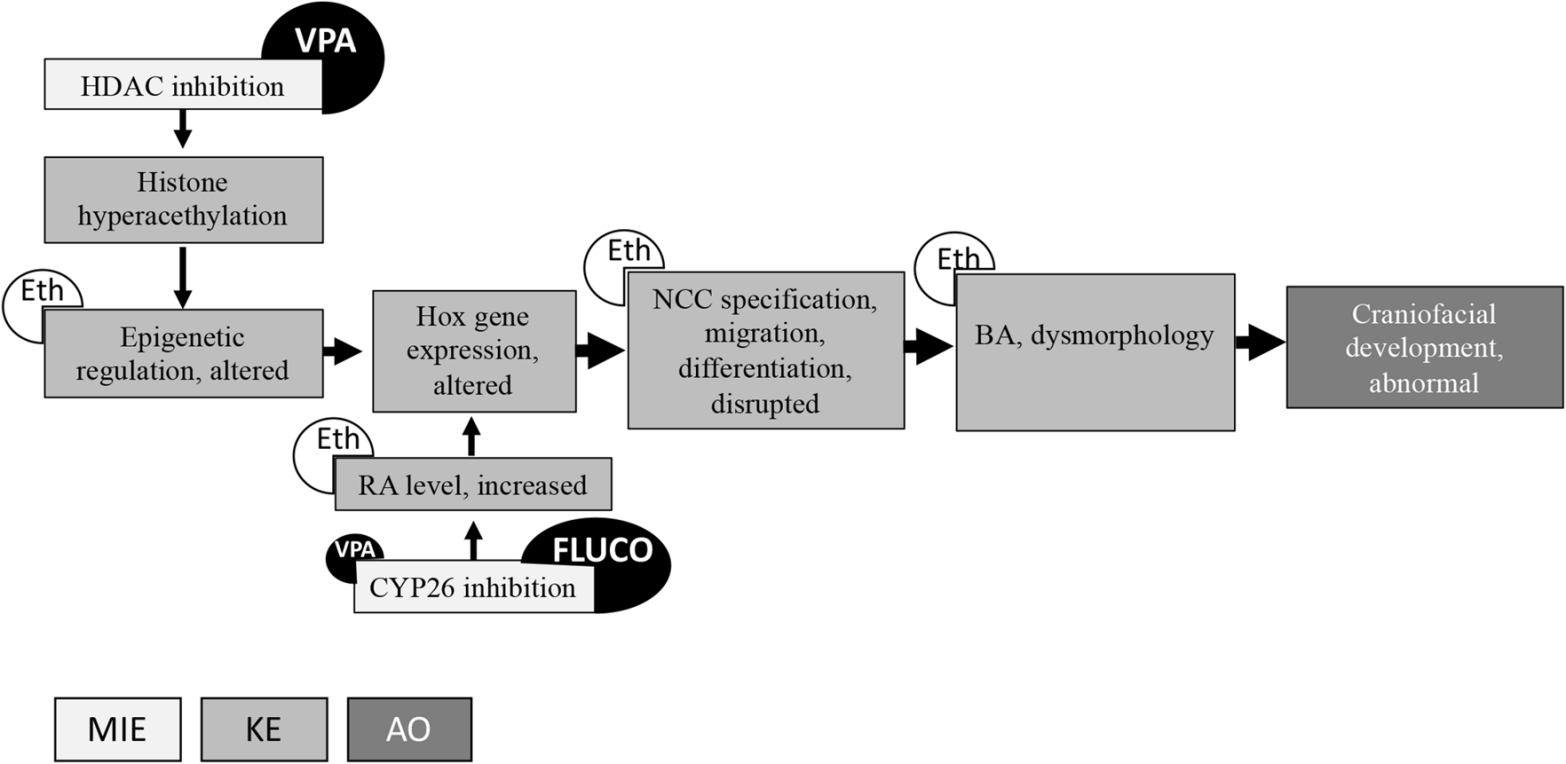
Schematic representation of the previously published AOP described for VPA and azoles (Metruccio et al. 2020; Menegola et al. 2021). FLUCO and VPA trigger different molecular initiating events (MIEs, light grey) inducing sequences of key events (KEs, grey) leading to the common adverse outcome (AO, dark grey). We propose an involvement of Eth (white circles) based on literature describing Eth role in different KEs of the above AOP

As far as a mechanistic approach is concerned, in the past our research group described the hypothetical adverse outcome pathway (AOP) for azole- and VPA-related facial defects (Metruccio et al. 2020; Menegola et al. 2021). AOP represents an information framework describing the progression of different toxicity events starting from one or more molecular initiating events (MIEs), that trigger a sequence of biological events (key events, KEs) finally leading to the final adverse outcome (AO) (Bal-Price and Meek 2017). The specific description of the previously published AOP includes two different MIEs (1. cytochrome CYP26 inhibition with consequent retinoic acid increase for azoles and with minor affinity for VPA; 2. histone deacetylase HDAC inhibition with consequent chromatin decondensation for VPA) and a number of KEs (Fig. 5). Literature on Eth-related pathogenic pathway leading to craniofacial defects describes some events already mentioned in our azole/VPA AOP (Fig. 5): (i) chromatin remodeling leading to altered epigenetic regulation (Liu et al. 2009; Mandal et al. 2017; Wallén et al. 2021), (ii) increased retinoic acid (Kane et al. 2010), (iii) neural crest cell specification- migration- differentiation disrupted (for a review see Smith et al. 2014), (iv) branchial arch dysmorphology (Giavini et al. 1992; van Maele-Fabry et al. 1995). We propose the involvement of Eth in enhancing one or more steps of the previously proposed AOP. This is consistent with our experimental results, showing mixture effects when embryos are co-exposed to Eth and FLUCO or VPA. Further ad hoc experiments are needed to improve knowledge and better describe the pathway as well as to evaluate Eth specific MIEs.

In conclusion, in the frame of chemical mixture effect evaluation, more attention should be paid on mild alcohol consumption in fertile women exposed to different molecules eliciting craniofacial alterations. The general recommendation of zero alcohol during pregnancy and pregnancy planning remains the safest strategy.
